# Can a List Experiment Improve Validity of Abortion Measurement?

**DOI:** 10.1111/sifp.12082

**Published:** 2019-01-23

**Authors:** Suzanne O. Bell, David Bishai

## Abstract

Although induced abortion is common, measurement issues have long made this area of research challenging. The current analysis applies an indirect method known as the list experiment to try to improve survey‐based measurement of induced abortion. We added a double list experiment to a population‐based survey of reproductive age women in Rajasthan, India and compared resulting abortion estimates to those we obtained via direct questioning in the same sample. We then evaluated list experiment assumptions. The final sample completing the survey consisted of 6,035 women. Overall, 1.8 percent of the women reported a past abortion via the list experiment questions, whereas 3.5 percent reported an abortion via the direct questions, and this difference was statistically significant. As such, the list experiment failed to produce more valid estimates of this sensitive behavior on a population‐based survey of reproductive age women in Rajasthan, India. One explanation for the poor list experiment performance is our finding that key assumptions of the methodology were violated. Women may have mentally enumerated the treatment list items differently from the way they enumerated control list items. Further research is required to determine whether researchers can learn enough about how the list experiment performs in different contexts to effectively and consistently leverage its potential benefits to improve measurement of induced abortion.

Elective pregnancy termination, or induced abortion, is a frequent reproductive health procedure that women throughout the world use to control their fertility. Current estimates indicate that approximately 56.3 (90 percent uncertainty interval [UI] 52.4 to 70.0) million induced abortions occurred annually between 2010 and 2014 (Sedgh et al. 2016). This corresponds to a global annual incidence of 35 induced abortions (90 percent UI 33 to 44) per 1,000 women aged 15–49 (Sedgh et al. 2016). Although induced abortion is common, measurement issues have long made this area of research challenging, particularly in low‐resource settings. Even in countries where abortion is broadly legal, facility data are often incomplete. In places where abortion is illegal or highly restricted, women often go outside the formal health sector and only present at formal sector facilities to receive post‐abortion care (PAC) for complications. The availability of safer self‐induction medicines, like misoprostol and mifepristone, has increased the measurement challenge associated with induced abortion, as women using safer methods may be less likely to present at facilities for PAC (Singh et al. 2010; Singh et al. 2018).

Relying instead on women's self‐report of abortion is subject to substantial underreporting as a result of social desirability pressure. A face‐to‐face interview is among the most common means of survey administration, but even in legal contexts like the United States, the pressure of social desirability results in less than 50 percent of abortions being reported (Jones and Kost 2007). Existing literature demonstrates that respondents are more willing to report sensitive behavior, like abortion, on self‐administered questionnaires (Lensvelt‐Mulders et al. 2005; Tourangeau and Yan 2007). However, in low‐resource and low‐literacy populations, trained enumerators typically administer surveys in a face‐to‐face setting. Audio computer‐assisted self‐interview (ACASI) often results in higher reporting of sensitive sexual behaviors than face‐to‐face interviews (Lara et al. 2004; Langhaug et al. 2011).

Asking about the sensitive item indirectly can reduce the impact of social desirability. Specifically, related to induced abortion, researchers have employed techniques such as randomized response technique (RRT) and the anonymous third‐party reporting (ATPR) method, as well as a modification of the ATPR referred to as the best friend method, with mixed but generally positive results (Elul 2004; Lara et al. 2004; Rossier et al. 2006; Coutts and Jann 2011; Yeatman and Trinitapoli 2011; Grossman et al. 2015). Additionally, researchers in India have used a mixed‐methods narrative approach to improve reporting with significant success (Edmeades et al. 2010). Each of these methods has a number of strengths and limitations but typically result in more valid estimates of induced abortion than direct questions, although there are some exceptions where results are more equivocal (Elul 2004; Fuentes 2017; Moseson, Gerdts, and Fuentes et al. 2017). Ultimately there is no gold standard for abortion measurement and the methodological choice is driven by the study context, the research objective(s), and the budget (Rossier 2003).

In India, induced abortion has been legal on request for a broad set of circumstances since the passage of the Medical Termination of Pregnancy Act of 1971. In Rajasthan, India specifically, official statistics from the Ministry of Health and Family Welfare indicate that 22,980 induced abortions were conducted in 2013, resulting in an annual induced abortion incidence of approximately 2 per 1,000 women aged 15–49 (Ministry of Health and Family Welfare 2013). However, these data are incomplete as they only include terminations conducted in certified facilities that are registered with the government to provide abortion. This excludes many private‐sector facilities as the process of registering with the government is cumbersome, while other providers are entirely unaware of this system. Results from a small study of women in Rajasthan revealed that 44 percent of women who reported a recent abortion had gone to a private‐sector doctor and 11 percent used the services of informal or untrained providers; these abortions would largely be unaccounted for in government statistics (Jejeebhoy 2011).

Government abortion service data also omit the substantial occurrence of self‐induction. A small survey of Rajasthani women conducted in 2001 found that nearly one in five women who reported a recent induced abortion had initially attempted self‐induction (Elul et al. 2004). The availability of misoprostol and mifepristone at pharmacies and chemists has only grown in recent years. Private‐sector drug distribution data indicate that the availability of misoprostol drug sales to wholesalers in India increased 646 percent from 2002 to 2007 (Fernandez et al. 2009). More recent estimates indicate that the volume of medical abortion drug sales is equivalent to approximately 34 abortions per 1,000 women aged 15–49 nationwide (Singh et al. 2018).

The current analysis applies an indirect method known as the list experiment to try to improve survey‐based measurement of induced abortion in Rajasthan, India. Social psychologists originally developed the list experiment method in the 1980s to elicit more truthful responses to sensitive questions (Miller 1984; Kuklinski et al. 1997; Sniderman and Carmines 1997). The standard list experiment randomizes individuals to either the treatment or control group. The control group is read a list of nonsensitive items, whereas the treatment group is read the same list, plus the sensitive item. Respondents are then asked to report *how many* of the items they have ever experienced (lifetime prevalence), not *which ones*, without directly mentioning each item. A simple difference in means between the mean total item counts of the treatment and control groups is then calculated. The double list experiment is a modification whereby every respondent receives a treatment version of one list and a control version of another list, thus everyone serves as control and treatment within the sample. This is a more efficient estimator than the standard list experiment.

For the list experiment to yield unbiased estimates of a given sensitive survey item, three assumptions must be met: (1) effective randomization, i.e., treatment and control groups are the same; (2) no design effect, i.e., addition of the sensitive item to the treatment list does not affect responses to the control items; and (3) honest responses (Blair and Imai 2012; Glynn 2013). Assumption 1 is under investigator control, whereas Assumptions 2 and 3 can be violated if respondents evaluate items on the list relative to one another or respond in a nonaccurate manner, either intentionally or not (e.g., due to misunderstanding).

In a list experiment, investigators can potentially overcome many of the challenges faced by other survey‐based approaches. Individuals can escape the social desirability pressures of direct questioning on a stigmatized item. The method requires limited additional training or cost if embedded in an existing survey. Also, there now exist multivariable analysis options for the list experiment (Corstange 2008; Imai 2011; Blair and Imai 2012; Glynn 2013). Researchers have increasingly used list experiments in place of RRT given that list experiments can be easier to implement and understand (Coutts and Jann 2011). Additionally, studies have shown that respondents trust and accept questions in the list experiment format more so than RRT (Coutts and Jann 2011). Results produced by the list experiment also have lower item nonresponse and can be more reliable than results from RRT, particularly for highly sensitive behaviors (Coutts and Jann 2011).

Most of the literature assessing this methodology compares list experiment estimates to those obtained via direct questioning or other survey methodologies. The general “more is better” assumption that higher estimates are more valid is typically used to assess performance (Tourangeau and Yan 2007). The assumption that a bigger estimate is more valid achieves consensus in settings where multiple strands of evidence suggest that direct questioning is leading to underreporting of stigmatized behaviors. Results from several empirical studies illustrate that the list experiment significantly outperforms direct questioning across multiple modes of administration, particularly with more sensitive behaviors (Tsuchiya et al. 2007; Gonzalez‐Ocantos et al. 2012; Comşa and Postelnicu 2013; Wolter and Laier 2014; Aronow et al. 2015). Specifically in the two face‐to‐face surveys that employed the list experiment (both of which were in low‐resource settings), list experiment estimates were higher than those obtained via direct questioning (Wolter and Laier 2014). In addition, interviewers reported greater comfort in asking the list experiment questions than the direct questions (Wolter and Laier 2014).

Despite these findings, which support use of a list experiment, there are several studies where list experiments failed to produce more valid estimates of the sensitive behavior(s) (Droitcour et al. 2004; Biemer and Brown 2005; Coutts and Jann 2011; Rosenfeld et al. 2016). A recent study using population‐level (as opposed to individual‐level) objective measures of the sensitive behavior found that, although list experiment estimates resulted in estimates that were higher than those obtained from direct questioning, RRT and endorsement experiment estimates were even higher (although the endorsement experiment confidence intervals were largest) (Rosenfeld et al. 2016). Further validation studies measuring a range of sensitive behaviors in different populations are needed to better understand when the list experiment is likely to outperform or underperform other methods.

In recent years, researchers have begun to assess the list experiment's performance specifically with regard to abortion measurement in a number of contexts with relative success (Moseson et al. 2015; Cowan et al. 2016; Moseson, Gerdts, Fuentes et al. 2017; Moseson, Treleaven, Gerdts et al. 2017; Treleaven et al. 2017). In a 2015 study measuring lifetime experience of abortion in Liberia, results indicated that 32 percent of women had ever had an abortion (Moseson et al. 2015). This list experiment estimate was five times greater than the only previous comparable estimate of induced abortion in the country, which had been estimated via direct survey questions six years prior. More recent research in the United States piloted list experiment questions using an online convenience sample of 1,233 women (Cowan et al. 2016). Twenty‐two percent and 18 percent of women reported a prior induced abortion in response to list experiment and direct questions, respectively; however, these estimates were not statistically significantly different (Cowan et al. 2016). Other unpublished work has also been conducted in Vietnam and Texas with mixed results (Moseson, Gerdts, Fuentes et al. 2017; Treleaven et al. 2017).

The current study brings methodological refinements that expand upon the typical analyses of list experiment abortion data. We added a double list experiment to a population‐based survey of reproductive age women in Rajasthan, India and compared resulting abortion estimates to those we obtained via direct questioning in the same sample. We then evaluated list experiment assumptions, providing additional evidence about the quality of the data collected from this technique in low‐resource settings. This work goes beyond other abortion‐specific list experiment applications by more thoroughly investigating list assumptions and hypothesized reasons for the observed performance of the list experiment. While most researchers have explored whether the randomization was effective in creating similar distributions of respondent characteristics across randomization groups, we further investigated another key list experiment assumption concerning design effects. Additionally, many existing applications of the list experiment to measure induced abortion do not have concurrently collected direct reports of abortion or do not fully utilize these data in exploring list experiment performance. Our inclusion of direct questions on prior abortion experience allows us to generate list experiment estimates by whether the respondent reported an abortion via direct questions. This enables partial investigation of the final list experiment assumption of honest responding. Taken together, this study contributes substantially to our understanding of list experiment performance in the context of abortion measurement.

## METHODOLOGY

### Data

In 2017 we added a double list experiment to a family planning survey of reproductive age women in Rajasthan, India conducted by researchers from the Bill & Melinda Gates Institute for Reproductive Health at the Johns Hopkins Bloomberg School of Public Health (JHSPH) and the Indian Institute of Health Management and Research (IIHMR). This survey was part of the Performance Monitoring and Accountability 2020 (PMA2020) project, which uses Open Data Kit (ODK) software on smart phones to routinely collect data on key family planning indicators every 6 to 12 months in 11 priority countries (Zimmerman et al. 2017). In each program country, a cadre of sentinel resident enumerators (REs) work in clusters to collect data at both the household and facility level. The platform is intended to measure progress toward achieving the Family Planning 2020 (FP2020) goal of providing contraception to 120 million additional women by the year 2020.

There was an initial PMA 2020 survey in Rajasthan in 2016. Data collection for the second round, which contained the list experiment and direct abortion questions, occurred in April and May of 2017. In preparation, investigators conducted a three‐day pilot with seven REs to adapt and finalize the list experiment and direct abortion questions and associated translations. During the pilot, we determined an appropriate and unequivocal Hindi translation for the phrase “had an induced abortion.” All REs then received a five‐day refresher training that reiterated important elements of survey implementation and the core family planning survey, and presented the new abortion material.

PMA2020/Rajasthan uses a probabilistic two‐stage cluster sampling design using urban/rural and region as the sampling domains and probability proportional to size sampling for the selection of enumeration areas (EAs) within sampling domains. Investigators took a random sample of 35 households from each of the 147 EA household sampling frames, which REs had created by mapping and listing EAs in Round 1. All eligible women, i.e., those aged 15–49 in selected households, were invited to participate in a brief interview related to sexual and reproductive health and past pregnancies. REs requested consent from all participants prior to administering the survey. The Institutional Review Boards (IRBs) at JHSPH and IIHMR provided ethical approval for the study.

We used the ODK form to randomize half of the respondents to Group 1, which received control list B (i.e., not including the sensitive item) followed by treatment list A (i.e., including the sensitive item) (Table 1). The other half of the respondents, Group 2, received control list A then treatment list B. We placed the list experiment questions in the first section of the survey following background questions to limit women's ability to determine the intent behind these questions. We then embedded the direct abortion questions in the reproductive history section following questions about previous births; this is consistent with the question order in India's National Family Health Survey (NFHS), allowing for maximum comparability of other contemporaneously collected direct abortion estimates. Putting the direct questions after the list experiment questions was also intended to eliminate the potential impact that answering a direct question on abortion might have on one's list experiment response.

**Table 1:** Double list experiment items

	List A	List B	Prevalence
Item 1	Had a menstrual period	Used a sanitary pad during a menstrual period	High
Item 2	Used contraceptive injections	Used a female condom	Low
Item 3^a^	Had an abortion	Had an abortion	
Item 4	Visited a health facility or camp	Been visited by an *anganwadi*, ASHA, or other community health worker	High
Item 5	Had a C‐section	Taken an ambulance to a hospital	Low

a Sensitive item; only added in treatment version of the list.

### Analysis

To derive a prevalence estimate from a list experiment we assume there is a population of *n* respondents randomly partitioned into *n*_1_ respondents who encountered the control list and *n*_2_ respondents who received the treatment list. Let $y_{Ai+}$ equal the total number of items that individual *i* reported ever experiencing from the treatment version of list A, which includes the sensitive item, and $y_{Ai-}$ equal the number of items that individual *i* reported ever experiencing from the control version of list A, which does not include the sensitive item. The estimated proportion ever experiencing the sensitive item (induced abortion) using list A, ${\hat{\pi }}_{A}$, is given by equation (1). A similar expression can be used for list B as seen in equation (2). These expressions allow one to calculate a difference in means between the average item count responses on the treatment and control versions of the lists.

(1) $${\hat{\pi }}_{A}=\frac{1}{n_{2}}\sum_{i=n_{1}+1}^{n}y_{Ai+}-\frac{1}{n_{1}}\sum_{i=1}^{n_{1}}y_{Ai-}$$

(2) $${\hat{\pi }}_{B}=\frac{1}{n_{1}}\sum_{i=1}^{n_{1}}y_{Bi+}-\frac{1}{n_{2}}\sum_{i=n_{1}+1}^{n}y_{Bi-}$$

Because we exposed each respondent to two lists in the double list experiment, we used the sample to produce two estimates of induced abortion prevalence using equations 1 and 2. We then took the average of these two induced abortion prevalence estimates for list A and B to produce an overall estimate.

The assumption of random partitioning of respondents was achieved through the ODK survey design. The assumption of no design effect, in other words that the addition of the sensitive item does not change an individual's response to the control items, can be represented mathematically. Using potential outcomes notation, we let $Z_{ij}\left(t\right)$ denote a binary variable that represents respondent *i*’s response for each control item *j* for *j* = 1,…, *J*, where *J* is the total number of control items on the list, under treatment status *t* = 0 for control list and 1 for treatment. Thus, for each *i* = 1,…, *N*, if there is no design effect, $\sum_{j=1}^{J}Z_{ij}\left(0\right)=\sum_{j=1}^{J}Z_{ij}\left(1\right)$.

The assumption that respondents are not falsifying their responses can also be represented mathematically, where the observed response of individual *i* for item *J*+1, the sensitive item, is assumed to be equal to the truthful answer to the sensitive question, i.e., $Z_{i,J+1}\left(1\right)=Z_{i,J+1}^{*}$, where $Z_{i,J+1}^{*}$ represents a truthful response to the sensitive item; $Z_{i,J+1}\left(0\right)$ is not defined since the sensitive item is not included in the control list.

The potential answer respondent *i* would give under the treatment and control conditions is denoted by $Y_{i}\left(0\right)=\sum_{j=1}^{J}Z_{ij}\left(0\right)$ and $Y_{i}\left(1\right)=\sum_{j=1}^{J+1}Z_{ij}\left(1\right)$, respectively, where $Y_{i}\left(1\right)$ is an integer between 0 and *J*+1 and $Y_{i}\left(0\right)$ is an integer between 0 and *J*. The observed response for respondent *i* is denoted $Y_{i}=Y_{i}\left(T_{i}\right)$ where $T_{i}$ denotes the treatment status actually assigned for a given list, 0 or 1.

The design effect and no falsified answers assumptions can be assessed by investigating the conditional probabilities of reporting *y* number of items depending on treatment assignment *T*, where the null hypothesis can be expressed as:

(3) PrYi≤y|Ti=0≥PrYi≤y|Ti=1 for  all y=0,…,J−1, and

Pr (Yi≤y|Ti=1)≥PrYi≤y−1|T1=0 for  all y=1,…,J.

If baseline responses are honest and respondents never overreport the sensitive behavior, then one can use a one‐sided test that the joint proportion, $\theta_{y}$, defined as $\theta_{y}=\mathrm{Pr}\left(Y_{i}\geq y|T_{i}=0\right)-\mathrm{Pr}\left(Y_{i}\geq y|T_{i}=1\right)$, is significantly different from zero in the negative direction. If the assumption of no design effect is satisfied, the addition of the sensitive item to the control list will make the response variable $Y_{i}$ in the treatment group larger than the control group response variable (the first line of equation (3)) but by no more than one item (the second line of equation (3)). If one of these joint proportions is negative, the assumption of no design effect (i.e., that the addition of the sensitive item to the control list does not affect an individual's response to the control items) is necessarily violated, as is the assumption of honest responses. Thus to assess whether the assumptions of no design effect and no falsified responses were violated, we conducted one‐sided t‐tests for the sample overall and by subgroup to determine if any of the $\theta_{y}$ were significantly less than 0 (Glynn 2013). If $\theta_{y}s$ are less than 0, one can re‐estimate the proportion experiencing the sensitive item by truncating the $\theta_{y}$ at 0 as seen in equation (4):

(4) θy=θy if θy≥0, and

θy=0 if θy<0

Then one can sum across the estimated $\theta_{y}s$ in the proportion reporting at least *y* items, which provides the piecewise list experiment estimate.

In this study, we first calculated the socioeconomic characteristics of the sample overall and by treatment assignment. We then generated list experiment and direct estimates of lifetime abortion experience overall and by age group, marital status, educational attainment, wealth quintile, caste, religion, residence (urban/rural), and parity. When calculating the overall and subgroup prevalence, we investigated and adjusted for violations of the aforementioned assumptions using the piecewise estimator previously described (Blair and Imai 2012; Glynn 2013).

To calculate the standard errors (SEs) associated with the list experiment estimates and to generate 95% confidence intervals (CIs) around the estimated difference between the list experiment and direct estimates, we used a resampling method. Specifically, we used the independent and identically distributed (iid) bootstrap with bias corrected CIs to account for potential non‐normality of the bootstrapped distribution of estimates (Efron 1987; Carpenter and Bithell 2000).

Given the sampling design, we employed a two‐stage resampling procedure to generate the sample distribution, accounting for the strata (urban/rural) and then selecting a random sample of *n* clusters (EAs) with replacement from the *n* sample clusters. The random sample of *m_i_* elements within the *i* ^th^ sample cluster was maintained, including all women within a given cluster each time it was randomly selected (StataCorp 2015a). Given that the number of units within clusters varied, the overall sample size across the resamplings also varied. For each of the samples, the survey weights, which accounted for the design weight and nonresponse, were normalized so that the average of the weights was always equal to 1.0. We resampled 1,000 times for each estimate, generating the sampling distribution of piecewise estimates for the list experiment as well as the difference between the piecewise estimates and the direct estimates, overall and by subgroup (Efron and Tibshirani 1994).

We conducted analyses in Stata version 15 and the R statistical platform, incorporating survey weights and accounting for the complex sampling design (R Development Core Team 2015; StataCorp 2015b).

## RESULTS

The final sample completing the survey consisted of 6,035 women. The response rate was 97.8 percent. Table 2 presents the characteristics of Rajasthani women aged 15–49. On average, women were 29 years old, the majority of whom (75.7 percent) were currently married or cohabiting. Large proportions of women had never attended school (36.8 percent), were of other backward classes (39.2 percent), were Hindu (85.3 percent), and resided in rural areas (64.2 percent). Nearly one‐third (31.1 percent) of women were nulliparous, while 36.1 percent had one to two children, and 24.7 percent had three to four children; only 8.2 percent had five or more children. Examining the characteristics of the women by group provides clear evidence that we achieved effective randomization, as anticipated.

**Table 2:** Socioeconomic characteristics of Rajasthani women aged 15–49, by group^a^

	Group 1		Group 2		Total	
	%	N	%	N	%	N
Mean age (SE)	29.3 (0.24)	2,989	28.6 (0.18)	3,046	28.9 (0.16)	6,035
Marital status						
Currently married/cohabiting	76.6	2,283	74.8	2,274	75.7	4,557
Divorced or separated/widowed	2.5	75	2.9	87	2.7	162
Never married	20.9	624	22.3	679	21.6	1,302
School						
Never attended	37.6	1,122	36.1	1,098	36.8	2,221
Primary	23.5	703	25.2	766	24.3	1,469
Secondary	17.5	522	17.6	537	17.6	1,059
Higher or postgraduate	21.5	641	21.1	644	21.3	1,285
Wealth						
Poorest	16.1	483	16.9	515	16.5	997
Second poorest	17.1	510	17.9	546	17.5	1,056
Middle	19.8	591	19.5	595	19.7	1,186
Second wealthiest	21.9	654	21.0	641	21.5	1,295
Wealthiest	25.1	751	24.6	750	24.9	1,500
Caste of household head						
Scheduled caste	22.2	663	22.4	683	22.3	1,346
Scheduled tribe	17.9	534	16.7	508	17.3	1,042
Other backward classes	38.4	1,146	39.9	1,216	39.2	2,362
General	21.5	642	20.9	637	21.2	1,279
Religion of household head						
Hindu	84.5	2,526	86.1	2,623	85.3	5,148
Muslim	13.9	415	12.7	386	13.3	801
Other	1.6	48	1.2	38	1.4	86
Residence						
Rural	64.3	1,920	64.1	1,954	64.2	3,874
Urban	35.7	1,068	35.9	1,092	35.8	2,160
Parity						
0	30.7	917	31.4	955	31.1	1,873
1–2	35.2	1,052	37.0	1,125	36.1	2,177
3–4	25.5	762	23.8	725	24.7	1,487
5+	8.5	254	7.9	239	8.2	493
Abortion (direct question)						
No	96.7	2,889	96.3	2,935	96.5	5,823
Yes	3.3	100	3.7	111	3.5	211
Total	100.0	2,989	100.0	3,046	100.0	6,035

a Estimates and Ns weighted.

Group 1 received control list B then treatment list A, Group 2 received control list A then treatment list B.

SE = Standard error.

Table 3 contains the overall list experiment estimate of lifetime experience of abortion among all women using the piecewise estimator, by list. The simple difference in means estimator generated an abortion prevalence of 0.2 percent and –1.4 percent on list A and B, respectively. Accounting for clear violations of the list experiment assumptions, namely a potential design effect whereby the joint proportion (Row 5) is negative, generated estimates of 2.5 percent and 1.1 percent on list A and B, respectively. As such, the overall piecewise estimate of lifetime prevalence of abortion was 1.8 percent (Table 3).

**Table 3:** List experiment estimates of lifetime experience of abortion among all Rajasthani women aged 15–49 using the piecewise estimator

		Number of reported items (proportion)						
	Source	0	1	2	3	4	5	Sum
**List A**								
Row 1	List with abortion	0.052	0.474	0.399	0.065	0.009	0.002	1.000
Row 2	Proportion at least	1.000	0.948	0.474	0.075	0.011	0.002	—
Row 3	List without abortion	0.042	0.470	0.429	0.055	0.004	0.000	1.000
Row 4	Proportion at least	1.000	0.958	0.488	0.059	0.004	0.000	—
Row 5	Row 2 minus Row 4	0.000	–0.010	–0.014	0.017	0.007	0.002	0.002
Row 6	Exclude violations	0.000	0.000	0.000	0.017	0.007	0.002	0.025
**List B**								
Row 1	List with abortion	0.115	0.529	0.277	0.068	0.008	0.002	1.000
Row 2	Proportion at least	1.000	0.885	0.356	0.078	0.010	0.002	—
Row 3	List without abortion	0.119	0.505	0.293	0.078	0.005	0.000	1.000
Row 4	Proportion at least	1.000	0.881	0.376	0.083	0.005	0.000	—
Row 5	Row 2 minus Row 4	0.000	0.004	–0.020	–0.004	0.005	0.002	–0.014
Row 6	Exclude violations	0.000	0.004	0.000	0.000	0.005	0.002	0.011
Average estimate across lists								–0.62%
Average estimate across lists, violations excluded								1.80%

NOTE: Rows 1 and 3 represent the proportions reporting each number of items on the treatment and control lists, respectively. Rows 2 and 4 represent the proportions reporting *at least* each number of items on the treatment and control lists, respectively. Row 5 represents the difference between Row 2 and 4, which is equal to the proportion of women who report having an abortion and the total number of treatment list items indicated by the column (i.e., the joint proportion). Row 6 is a replicate of Row 5 where negative estimates have been excluded. The sum across columns for Row 5 and 6 represent the overall estimate of the proportion of women reporting a past abortion, including and excluding violations (i.e., negative joint proportions in Row 5), respectively.

— = Sum not applicable.

Table 4 presents the list experiment abortion prevalence estimates overall and by background characteristics along with the direct abortion estimates. The direct and list experiment estimates were similar within some background characteristics. For instance, abortion prevalence increased with age, peaking at 30–39 before reducing among the oldest cohort of women. Prevalence estimates generated via direct and list experiment questions increased with increasing education but declined slightly among those with higher or postgraduate education. Hindu women reported the lowest experience with abortion, while Muslim women reported slightly higher levels of abortion and other religions’ estimates were higher still. Abortion estimates were higher among urban compared to rural women.

**Table 4:** Estimate of lifetime experience of induced abortion among Rajasthani women aged 15–49, by socioeconomic characteristics and measurement methodology^a^

	Direct	List Experiment	List Experiment–Direct
	% (SE)	% (SE)	% (95% CI)^b^
Age			
15–19	0.2 (0.1)	0.7 (1.0)	0.5 (–0.2, 1.7)
20–29	3.9 (1.0)	4.3 (1.6)	0.4 (–2.4, 3.1)
30–39	5.7 (0.9)	5.3 (1.9)	–0.4 (–4.3, 2.6)
40–49	2.8 (1.0)	1.2 (1.3)	–1.6 (–3.2, 0.4)
Marital status			
Currently married/cohabiting	4.6 (0.9)	2.2 (1.0)	–**2.4 (**–**3.9,** –**1.1)**
Divorced or separated/widowed	1.4 (1.1)	3.4 (5.1)	1.9 (–1.3, 2.9)
Never married	0.1 (0.1)	0.7 (0.9)	0.6 (–0.3, 1.5)
School			
Never attended	2.8 (0.6)	2.2 (1.6)	–0.6 (–2.8, 1.8)
Primary	3.4 (0.8)	2.4 (1.1)	–1.0 (–3.5, 1.2)
Secondary	4.4 (1.4)	5.5 (2.3)	1.2 (–3.6, 5.5)
Higher or postgraduate	4.2 (1.0)	2.3 (1.8)	1.9 (–4.4, 1.1)
Wealth			
Poorest	1.5 (0.6)	2.2 (1.4)	0.7 (‐1.3, 3.1)
Second poorest	1.3 (0.4)	2.6 (1.7)	1.3 (–0.9, 3.2)
Middle	3.6 (1.0)	2.7 (1.3)	–0.9 (–3.3, 0.8)
Second wealthiest	4.6 (1.5)	2.4 (1.6)	–**2.2 (**–**5.0,** –**0.4)**
Wealthiest	5.3 (1.3)	2.1 (1.7)	–**3.3 (**–**7.2,** –**1.3)**
Caste of household head			
Scheduled caste	3.6 (1.1)	2.4 (1.7)	–1.3 (–4.3, 0.7)
Scheduled tribe	3.6 (1.4)	6.6 (2.3)	2.9 (–1.6), 7.3)
Other backward classes	3.0 (0.9)	2.0 (1.3)	–1.0 (–3.4, 1.1)
General	4.2 (1.1)	4.1 (1.8)	–0.1 (–5.1, 2.7)
Religion of household head			
Hindu	3.3 (0.6)	1.9 (0.9)	–1.4 (–3.4, 0.2)
Muslim	4.4 (2.1)	3.4 (3.8)	–1.0 (–8.1, 1.8)
Other	9.0 (3.1)	12.4 (40.0)	3.4 (–10.6, 97.1)
Residence			
Rural	1.9 (0.4)	1.3 (0.7)	–0.6 (–1.9, 0.7)
Urban	6.4 (1.7)	3.6 (1.8)	–**2.9 (**–**7.0,** –**0.5)**
Parity			
0	0.2 (0.1)	1.2 (1.0)	0.9 (–0.1, 2.1)
1–2	5.5 (1.1)	3.2 (1.4)	–2.3 (–5.8, 0.4)
3–4	4.8 (1.0)	2.1 (1.7)	–1.8 (–4.7, 0.8)
5+	3.2 (1.1)	6.6 (3.1)	3.4 (–2.9, 8.0)
Total	3.5 (0.7)	0.0 (0.8)	–**1.7 (**–**3.3,** –**0.5)**

a Estimates weighted. Bolding indicates p‐value less than 0.05.

b Bias‐corrected bootstrapped 95% confidence intervals.

However, Table 4 shows that direct and list experiment estimates diverged within other background characteristics. For example, the direct estimates of abortion were higher for currently married or cohabiting women while list experiment estimates were slightly higher for divorced, separated, or widowed women. Direct abortion estimates increased with increasing wealth, while list experiment estimates remained similar across wealth quintiles; estimates by caste and parity also followed different patterns.

When comparing the direct and list experiment estimates quantitatively, very few estimates were statistically significantly different (Table 4). Overall, the list experiment abortion prevalence estimate was 1.7 percent lower than the direct estimate, which was statistically significant (p<0.01). The only subgroup estimates that were significantly different by method were currently married or cohabiting women (2.4 percent, p<0.01), the second wealthiest (2.2 percent, p<0.05) and wealthiest (3.3 percent, p<0.05), and urban women (2.9 percent, p<0.05), all of which had significantly higher direct abortion estimates.

In examining the list experiment assumptions, potential explanations for its poor performance begin to be revealed. While effective randomization was achieved, results from the assessment of design effects demonstrated clear violations of the list experiment assumption, and likely the assumption of honest responding. Table 5 contains the p‐values for the associated design effect significance test overall and by background characteristics for each list. For list A, there was evidence of significant design effects only for those who never attended school and those from other backward classes. However, list B had more violations of the design effect assumption, with statistically significant violations detected among women aged 15–19, those from a scheduled tribe, Hindu women, nulliparous women, and women who reported no past experience with abortion via the direct questions.

**Table 5:** Detection of list experiment design effect violations by socioeconomic characteristic and list among Rajasthani women aged 15–49^a^

	Design Effect P‐Value	
	List A	List B
Age		
15–19	0.208	**0.001**
20–29	0.545	0.562
30–39	0.271	0.661
40–49	0.653	0.423
Marital status		
Currently married/cohabiting	0.645	0.567
Divorced or separated/widowed	0.523	1.000
Never married	0.450	0.079
School		
Never attended	**0.043**	0.998
Primary	0.557	0.222
Secondary	0.171	0.061
Higher or postgraduate	0.812	0.696
Wealth		
Poorest	0.318	0.260
Second poorest	0.912	0.509
Middle	0.473	0.428
Second wealthiest	0.063	0.701
Wealthiest	0.836	0.557
Caste of household head		
Scheduled caste	0.380	0.814
Scheduled tribe	0.527	**0.017**
Other backward classes	**0.002**	1.000
General	1.000	0.171
Religion of household head		
Hindu	0.574	**0.035**
Muslim	0.550	0.433
Other	0.281	0.289
Residence		
Rural	0.901	0.149
Urban	0.071	0.961
Parity		
0	0.501	**0.003**
1–2	0.596	0.219
3–4	0.125	1.000
5+	0.776	1.000
Abortion (direct question)		
No	0.513	**0.009**
Yes	0.320	0.996
Total	0.531	0.119

a Each list/subgroup specific p‐value is Bonferroni‐corrected to account for multiple comparison within the design effect test. Bolding indicates p‐value less than 0.05.

Investigating the list experiment performance further by whether women reported an abortion via the direct questions provides additional insights (Tables 6a and 6b). Among women who reported having an abortion on the direct questions, the list experiment estimate of abortion prevalence was 41.1 percent among those who received treatment list A and 94.5 percent among those who received treatment list B; the average estimate was 67.8 percent (Table 6a). This provides clear evidence that women who had an abortion were actually *less* likely to include the experience in their numeric response to the list experiment questions than they were on the direct questions asked later in the survey; this was particularly true for list A. Note that there were no negative joint proportions (Table 6a, Rows 5) in the piecewise estimates among women with a known abortion, thus there were no design effects among this subgroup.

**Table 6a:** List experiment estimate of lifetime experience of abortion using the piecewise estimator among Rajasthani women aged 15–49 who reported abortion in direct question

		Number of reported items (proportion)						
	Source	0	1	2	3	4	5	Sum
**List A**								
Row 1	List with abortion	0.000	0.152	0.454	0.331	0.050	0.014	1.000
Row 2	Proportion at least	1.000	1.000	0.848	0.394	0.063	0.014	—
Row 3	List without abortion	0.008	0.202	0.665	0.125	0.000	0.000	1.000
Row 4	Proportion at least	1.000	0.992	0.790	0.125	0.000	0.000	—
Row 5	Row 2 minus Row 4	0.000	0.008	0.058	0.269	0.063	0.014	0.411
Row 6	Exclude violations	0.000	0.008	0.058	0.269	0.063	0.014	0.411
**List B**								
Row 1	List with abortion	0.013	0.146	0.460	0.288	0.094	0.000	1.000
Row 2	Proportion at least	1.000	0.987	0.841	0.382	0.094	0.000	—
Row 3	List without abortion	0.091	0.550	0.268	0.091	0.000	0.000	1.000
Row 4	Proportion at least	1.000	0.909	0.359	0.091	0.000	0.000	—
Row 5	Row 2 minus Row 4	0.000	0.078	0.482	0.291	0.094	0.000	0.945
Row 6	Exclude violations	0.000	0.078	0.482	0.291	0.094	0.000	0.945
Average estimate across lists								67.79%
Average estimate across lists, violations excluded								67.79%

NOTE: Rows 1 and 3 represent the proportions reporting each number of items on the treatment and control lists, respectively. Rows 2 and 4 represent the proportions reporting *at least* each number of items on the treatment and control lists, respectively. Row 5 represents the difference between Row 2 and 4, which is equal to the proportion of women who report having an abortion and the total number of treatment list items indicated by the column (i.e., the joint proportion). Row 6 is a replicate of Row 5 where negative estimates have been excluded. The sum across columns for Row 5 and 6 represent the overall estimate of the proportion of women reporting a past abortion, including and excluding violations (i.e., negative joint proportions in Row 5), respectively.

— = Sum not applicable.

**Table 6b:** List experiment estimate of lifetime experience of abortion using the piecewise estimator among Rajasthani women aged 15–49 who did not report abortion in direct question

		Number of reported items (proportion)						
	Source	0	1	2	3	4	5	Sum
**List A**								
Row 1	List with abortion	0.054	0.485	0.397	0.055	0.008	0.001	1.000
Row 2	Proportion at least	1.000	0.946	0.461	0.064	0.009	0.001	—
Row 3	List without abortion	0.043	0.480	0.420	0.052	0.004	0.000	1.000
Row 4	Proportion at least	1.000	0.957	0.477	0.056	0.004	0.000	—
Row 5	Row 2 minus Row 4	0.000	–0.010	–0.015	0.008	0.005	0.001	–0.011
Row 6	Exclude violations	0.000	0.000	0.000	0.008	0.005	0.001	0.014
**List B**								
Row 1	List with abortion	0.119	0.544	0.270	0.060	0.005	0.002	1.000
Row 2	Proportion at least	1.000	0.881	0.337	0.067	0.007	0.002	—
Row 3	List without abortion	0.120	0.503	0.294	0.077	0.005	0.000	1.000
Row 4	Proportion at least	1.000	0.880	0.377	0.082	0.005	0.000	—
Row 5	Row 2 minus Row 4	0.000	0.001	–0.039	–0.016	0.002	0.002	–0.051
Row 6	Exclude violations	0.000	0.001	0.000	0.000	0.002	0.002	0.004
Average estimate across lists								–3.08%
Average estimate across lists, violations excluded								0.95%

NOTE: Rows 1 and 3 represent the proportions reporting each number of items on the treatment and control lists, respectively. Rows 2 and 4 represent the proportions reporting *at least* each number of items on the treatment and control lists, respectively. Row 5 represents the difference between Row 2 and 4, which is equal to the proportion of women who report having an abortion and the total number of treatment list items indicated by the column (i.e., the joint proportion). Row 6 is a replicate of Row 5 where negative estimates have been excluded. The sum across columns for Row 5 and 6 represent the overall estimate of the proportion of women reporting a past abortion, including and excluding violations (i.e., negative joint proportions in Row 5), respectively.

— = Sum not applicable.

Among women who reported no abortion on the direct questions, women were again less likely to report this experience via the list experiment, resulting in a difference in mean estimate of –1.1 percent and –5.1 percent for list A and B, respectively (Table 6b, Rows 5). Results from the piecewise estimator generated estimates of 1.4 percent and 0.4 percent on list A and B, respectively, which average to 0.9 percent. Thus, the list experiment identified a very small proportion of women who ever had an abortion but who denied it on the direct abortion questions only after accounting for design effect violations.

Additionally, we conducted a number of sensitivity analyses to explore the role of RE‐respondent acquaintance in explaining the poor performance of the list experiment. Bivariate results indicate that the list experiment and direct abortion question estimates were generally higher when the RE was less acquainted with the respondent, but estimates were not statistically significantly different by acquaintance; this was true when exploring the four‐category acquaintance variable (very well acquainted, well acquainted, not well acquainted, and not at all acquainted) as well as a dichotomous acquaintance variable where we combined the first two categories and the second two categories of acquaintance (results not shown). The list experiment only produced higher abortion estimates than the direct questions among respondents not at all acquainted with the interviewer when using the four‐category acquaintance variable, but the difference did not reach statistical significance. The direct question estimates were statistically significantly higher than the list estimates for the not‐well‐acquainted group when examining the four‐category variable, as well as the acquainted group when examining the dichotomous variable. Thus, no clear evidence emerged to suggest that the poor performance of the list experiment was due to RE‐respondent familiarity.

## DISCUSSION

The overall abortion prevalence estimate from our list experiment was lower than our direct questions estimate. In total, 1.8 percent of women reported a past abortion via the list experiment questions whereas 3.5 percent reported an abortion via the direct questions and this difference was statistically significant. As such, the list experiment failed to produce larger estimates of this sensitive behavior on a population‐based survey of reproductive age women in Rajasthan, India. Given a widely accepted assumption that abortion estimates obtained via direct questions are underreports, the list experiment estimates lack validity. Estimates within subgroups were generally similar across methodologies, but the direct abortion estimates were significantly higher among currently married or cohabiting women, wealthier women, Hindu women, and urban women.

These results were not entirely unexpected given that recent applications of the list experiment to measure aspects of induced abortion in low‐resource settings have generated mixed results. The list experiment performed well in the initial Liberian application that measured lifetime experience of abortion (Moseson et al. 2015), while it produced lower than expected sex selective abortion estimates in Vietnam (Treleaven et al. 2017). Additionally, application of the list experiment produced only slightly higher estimates in a United States online survey (Cowan et al. 2016) and much higher than anticipated estimates of self‐induced abortion in Texas (Moseson, Gerdts, Fuentes et al. 2017). We should also note that attempts to measure induced abortion *incidence* via a double list experiment on our survey of Rajasthani women failed to produce a positive incidence estimate using the difference in means calculation (–34.1 abortions per 1,000 women aged 15–49), but the piecewise estimated abortion incidence was 15.8 abortions per 1,000 women of reproductive age (95% CI 5.0–28.1); this was significantly higher than the direct abortion incidence estimate of 4.1 per 1,000 (95% CI 1.8–6.5). Taken together, these findings cast doubt on the validity of list experiment measures of abortion in a low‐resource setting.

In the context of the PMA2020/Rajasthan survey, one obvious explanation for the poor list experiment performance is our evidence that list assumptions were violated. Women may have mentally enumerated the treatment list items differently from the way they enumerated control list items. This presentation of a design effect was partially accounted for in the piecewise estimator, but the associated estimates could not fully adjust for this behavior. Ceiling or floor effects among women who would have either responded with the highest number of items on the treatment list (5) or the lowest (0) may have contributed to the observed design effect. Alternatively, women may have simply omitted abortion in giving their treatment list response regardless of how many control items they were reporting.

There are several other potential explanations for why the list experiment failed to produce improved estimates of induced abortion. The list experiment may simply be too cognitively demanding for respondents. This may lead respondents to provide spurious answers to the list experiment questions. Alternatively, poor cognitive ability or numeracy may have resulted in women incorrectly providing their response by indicating the specific items they had experienced, which is a clear violation of the confidentiality that this method is meant to afford. Finding that our direct and list experiment estimates of abortion by education were not statistically significantly different argues against a large role for cognitive limitations in distorting the size of the estimates. However, there *was* evidence of a statistically significant design effect among the subgroup of respondents who had never attended school. So poor cognitive ability did play some role in response generation, but not enough to make list estimates outperform direct estimates in any educational subgroup.

Beyond potential poor understanding of the list experiment questions and associated instructions, women may have not interpreted the sensitive item, or their corresponding past behavior, accurately. To the extent that a woman does not view a past experience as an abortion, she will correctly not include that experience in her answer to questions about abortion on surveys (Simonds et al. 1998; Kanstrup et al. 2017). Relatedly, our phrasing of induced abortion may have been too narrow for the Indian context. Evidence from other settings highlight the experience of “bringing back one's period,” similar to menstrual regulation (Plummer et al. 2008; Rahman et al. 2014). Using only the phrase “had an induced abortion” in our survey may help to explain the low direct and list experiment estimates, but since the same term was used for both list and direct estimates, the terminology would not explain why list estimates were lower than direct estimates. Future use of a list experiment may benefit from exploring the use of a more encompassing item description that includes reference to both induced abortion and period regulation.

Additionally, given the placement of the list experiment at the beginning of the survey and the direct questions later, more rapport between REs and respondents may have developed between when REs asked the list experiment questions and the direct questions (Sudman et al. 1996). As such, women may have felt more comfortable revealing their abortion later in the survey, or the initial exposure to the topic of abortion in the list experiment cognitively primed the respondents (Sudman et al. 1996). Last, the poor list experiment performance could simply be a result of poor implementation on the part of the REs or poor engagement on the part of the respondents. Further research could explore some of these proposed explanations for the list experiment's failure to produce improved estimates of abortion prevalence or incidence in this sample of Rajasthani women.

This study has a number of strengths. The data collected provide a large, representative sample of reproductive age Rajasthani women. The female questionnaire included both direct and list experiment questions on abortion, providing an in‐sample contemporaneous comparison of the two methodologies. The large sample size provided sufficient power to detect significant differences across subgroups of background characteristics. Additionally, interviewers were largely *resident* enumerators, meaning that the interviewer had the potential added advantage of being from the area of most of her respondents. This may have improved survey implementation and translation into local languages, and existing literature suggests it may have helped to create an environment in the survey interaction that increased respondents’ willingness to reveal sensitive behaviors (Weinreb 2006; Rodriguez et al. 2015; Sana et al. 2016).

Despite these strengths, this investigation had a number of limitations. These limitations present opportunities for improvement in future list experiment implementations and we present them as a practical set of lessons learned. Our list experiment design used four control items, but we recommend trying fewer control items. Including fewer control items on the list(s) may minimize the cognitive demand associated with answering the questions, which could be especially important when implementing in a low‐literacy setting. The success of the list experiment in Liberia may be partially attributable to their use of only three control items (Moseson et al. 2015). We also recommend doing extensive testing of different control items to identify the best performing control lists. We generated the control lists in conjunction with our in‐country partners and made several modifications during the pilot, but more extensive piloting of potential control items may have led to improved list experiment performance and less variation in performance across lists. Additionally, we highly recommend conducting qualitative cognitive interviews during the pilot to better understand how the respondents are interpreting and mentally enumerating the list experiment and the individual items. We only added quantitative cognitive interview questions at the end of the pilot questionnaire, limiting our ability to determine respondents’ full understanding of the list experiment questions and whether they knew the list experiment design protected the confidentiality of their responses.

We encourage the use of a dummy list following the list experiment instructions to ensure the respondent knows to provide only a numeric response, which we did using a list of local foods. Even better would be the use of a dummy list that measures something innocuous that is measured directly elsewhere in the survey. This will determine whether the list experiment can effectively be used to measure any item accurately in the given survey context, thus revealing whether a failure of the abortion list experiment questions is due to the sensitive nature of abortion or poor performance of the list experiment more generally.

Regarding training, adequate list experiment training time must be scheduled regardless of interviewers’ prior survey participation. The REs we trained were not professional interviewers and 38.5 percent of REs indicated on a post‐data collection survey that they experienced difficulty implementing the list experiment questions as intended. Additional training may have mitigated this difficulty. Last, we had no external estimates against which to validate the direct or list experiment results. However, we recommend including direct questions for comparison as we did.

Every survey‐based study faces similar challenges of optimizing syntax and ensuring consistent, clear survey administration. List experiments amplify these challenges. The formative work to develop the training and wording of this survey was extensive. Nevertheless, it is impossible to know whether having a longer pilot or training would have affected the validity of the list experiment estimates in this context. One clue that training would not have helped was that there did not appear to be a subset of interviewers for whom the list experiment results were producing the expected improvement in abortion measurement.

Further examination is required to determine contexts and conditions in which application of a list experiment is most likely to be beneficial and result in improved abortion estimates. A recent publication more thoroughly summarizes best practices and remaining questions regarding using a list experiment to measure induced abortion (Moseson, Treleaven, Gerdts et al. 2017). We encourage those who recently used or are planning to use a list experiment to publish their findings regardless of the list experiment's performance. Subsequently conducting a meta‐analysis of list experiment performance in measuring abortion and other sensitive behaviors will allow advancement of the science around this methodology, and failures of the list experiment, like this one, must be represented. Time will tell whether researchers can learn enough about the list experiment to effectively and consistently leverage its potential benefits to improve measurement of induced abortion, or whether the methodology will lose appeal.
